# Congenital Long QT Syndrome: A Focus on Risk Stratification and Management

**DOI:** 10.31083/RCM36779

**Published:** 2025-06-27

**Authors:** Deepti Ranganathan, Steffany Grondin, Raouane Hadjeres, Jacqueline Joza

**Affiliations:** ^1^Department of Cardiology, McGill University Health Centre, Montreal, QC H4A 3J1, Canada

**Keywords:** long QT syndrome, QT prolongation, risk stratification

## Abstract

Congenital long QT syndrome (LQTs) is an inherited cardiac condition resulting from cardiac repolarization abnormalities. Since the initial description of congenital LQTs by Jervell and Lange-Nielsen in 1957, our understanding of this condition has increased dramatically. A diagnosis of congenital LQTs is based on the medical history of the patient, alongside electrogram features, and a genetic variant that is identified in approximately 75% of cases. The appropriate risk stratification involves a multitude of factors, with β-blockers being the cornerstone of therapy. Recent developments, such as the incorporation of artificial intelligence (AI) for electrocardiogram (ECG) interpretation, genotype–phenotype-specific therapies, and emerging gene therapies, may potentially make personalized medicine in LQTs a reality in the near future. This review summarizes our current understanding of congenital LQTs, with a focus on risk stratification, current therapeutic interventions, and emerging developments in the management of congenital LQTs.

## 1. Introduction

Congenital Long QT syndrome (LQTs), first described by Jervell and Lange-Nielsen in 1957, is an inherited cardiac channelopathy characterized by repolarization abnormality, characterized as prolonged QT interval on electrocardiogram (ECG) [1]. This condition predisposes individuals to recurrent syncope and fatal arrhythmias such as torsade de pointes (TdP) and sudden cardiac death [1]. Since its initial description, significant advancements have been made in understanding the underlying genetic predisposition, clinical presentation and management of this condition.

Disease-causing gene mutations have been identified in 75% of cases of LQTs and are further divided into subtypes based on the underlying genetic disruption [2]. Genes encoding for cardiac ion channels, with *KCNQ1*, *KCHN2*, *SCN5A* represent most of the common pathogenic variants in LQTs subtypes 1,2 and 3 respectively. Despite the growing insights into genotype-phenotype correlation for gene-specific therapies in LQTs, varying degrees of penetrance and phenotypic expression pose a significant diagnostic challenge [3]. Furthermore, optimising risk reduction in LQTs remains a concern. Recent developments such as incorporation of artificial intelligence (AI) for ECG interpretation, genotype-phenotype specific therapies, and emerging gene therapies can potentially make personalized medicine in LQTs possible in the near future.

This review aims to provide an up-to-date synthesis of the current understanding of LQTs, focusing on diagnosis, risk stratification, current and novel tools for therapeutic developments in the management of patients with LQTs.

## 2. Diagnosis

Table 1 outlines the recently proposed modified LQTs score 1 [4]. A clinical diagnosis of LQTs is established when the score is >3 or when repeated ECGs show a corrected QT(QTc) interval of ≥480 msec regardless of symptoms. A QTc interval ≥460 msec is sufficient for diagnosing LQTs in the presence of symptoms [4].

**Table 1.** **Modified LQTs diagnostic score**.

Finding			Points
History			
	Clinical history of syncope		
		∙ Without stress	1
		∙ With stress	2
Family history			
	Family history of definite LQTs		1
	Unexplained sudden death in a first-degree family member <30 years		0.5
ECG			
	Correct QT interval (QTc interval by Bazett’s formula: QT/√RR)		
		∙ 450–459 msec (in males)	1
		∙ 460–479 msec	2
		∙ ≥480 msec	3.5
	QTc interval increase ≥480 msec at 4th min recovery from the exercise stress test		1
	Torsade de pointes		2
	T-wave alternans		1
	>3 leads with notched T-waves		1
	Bradycardia for age		0.5
Genetic finding			
	Pathogenic variant identification		3.5

LQTs, long QT syndrome; QTc, corrected QT; RR, time between 2 successive R waves on ECG; ECG, electrocardiogram.

It is important to note that adult women have longer QTc intervals than men. Although the mechanism of gender difference seen in human ECGs are not fully understood, it is believed that sex hormones, testosterone and progesterone, influence the repolarisation complex [5]. The changes in sex hormones during the menstrual cycle, pregnancy and menopause have a complex effect on the QTc interval in women but remain poorly understood [5]. Moreover, it is crucial to acknowledge that the response to treatment, particularly β-blockers, differs between males and females, with males exhibiting a more significant shortening of the QTc interval following medication administration [6].

QTc measurement remains the cornerstone of the diagnosis of LQTs. However, despite its apparent simplicity, incorrect QTc measurement leads to erroneous diagnoses, significantly influencing the potential for diagnostic reversals in affected patients. A prior study reported that 62% of electrophysiologists and <25% of cardiologists and non-cardiologists accurately identified QTc intervals as being either “long” or “normal” [7]. A recent study conducted by the Mayo Clinic’s specialised LQTs clinic reported a total of 451 patient-years of unwarranted medical therapy, with 8% of the study population inappropriately receiving implantable cardioverter-defibrillators (ICDs) as a result of overdiagnosis [8]. The inclusion of U waves in the measurement, as well as physician adjudication of the QTc interval intervals as ‘borderline’, were some of the most commonly reported reasons for overdiagnosis of LQTs.

It is important to highlight that in approximately one in four patients with LQTs, the QTc interval can be normal, a phenomenon referred to as ‘concealed’ LQTs [9]. As such, it is critical to unmask QT changes with dynamic testing.

Initial studies by Bazett showed that an abrupt increase in heart rate results in acute shortening of the action potential after the first fast heartbeat but requires several hundred beats or up to 2 minutes before a new steady state is achieved [10, 11, 12]. Patients with LQTs have been shown to have a maladaptive response to changes in heart rate, especially when this occurs suddenly, most notably in patients with LQT 2 [13, 14]. The epinephrine challenge is no longer recommended given high interobserver and intraobserver interpretation resulting in poor reproducibility and reliability [15]. Other methods that induce sudden changes in heart rate, such as exercise stress testing, hyperventilation and rapid standing with ECG monitoring, can provide valuable and practical diagnostic information [16]. Resultant sinus tachycardia from adrenergic stimulation affects QTc interval independently of the concomitant tachycardia [17]. A QTc interval ≥480 msec at the 4-minute recovery ECG during exercise testing has been included as part of the modified Schwartz score for LQTs [4]. In LQT 2, the notch noted on T-waves, also becomes more prominent in recovery. Other studies have also shown the usefulness of exercise-induced repolarisation parameters that aid in the diagnosis, such as the 1-minute post-brisk standing from sitting ECG [18, 19, 20].

**Fig. 1.**
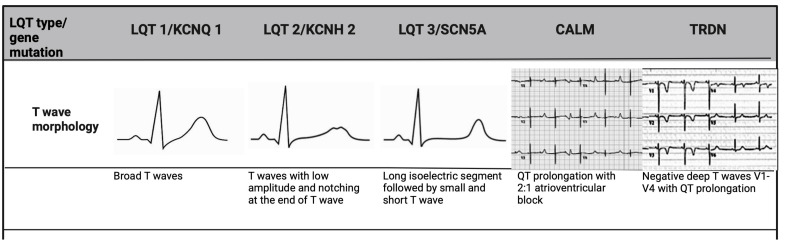
**T-wave morphology specific to LQTs genotype**. LQT 1 shows broad-based T-waves; LQT 2 demonstrates low-amplitude and bifid T-waves; and LQT 3 presents with a prolonged ST-segment and a late-peaking T-wave. CALM mutations, manifest earlier in life, and are associated with bradycardia and atrioventricular block, while TRDN mutation shows deeply inverted T-waves in precordial leads. LQTs, long QT syndrome; TRDN, triadin.

When QTc interval duration is borderline, the repolarisation morphology of T-waves on ECGs are particularly useful not only in making the diagnosis, but also in differentiating subtypes of LQTs [21]. In LQT 2, a distinct bifid T-wave morphology is noted, whereas LQT 3 will show an iso-electric interval before a low amplitude T-wave, similar to that seen in hypocalcaemia. In LQT 1, the T-wave morphology appears more broad-based and prolonged (Fig. 1). Given that these changes are subtle in LQT patients, it is imperative to assess all 12 leads of the ECG [16].

## 3. Genetic Considerations

**Fig. 2.**
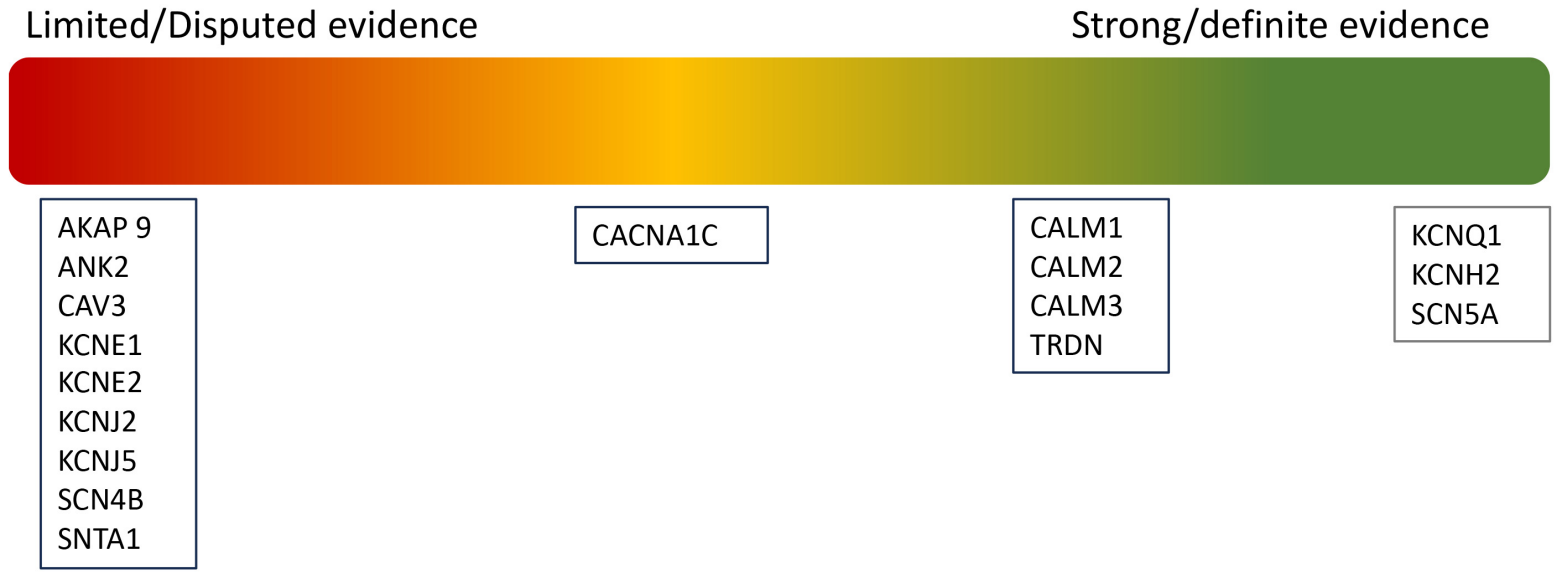
**Re-classification of pathogenic variants in LQTs**.

Although 17 genes were initially identified as causative of LQTs, a recent reappraisal by ClinGen has led to a reclassification, only seven genes now considered to have strong or definitive evidence for LQTs [22], as summarised in Fig. 2.

The majority of cases of LQTs are caused by loss-of-function variants in voltage-gated potassium channels [23]. The two subtypes of delayed rectifier potassium channels, K_V_7.1 (slow) and K_V_11.1 (rapid) are primarily responsible for the outward potassium current in phase 3 of the ventricular action potential. These channels play a crucial role in cardiac myocardial repolarisation [23, 24]. LQT 1 is caused by a variation in *KCNQ1* that encodes for the ɑ subunit of K_V_7.1, while LQT 2 is caused by a variation in *KCNH2*, which encodes for the ɑ subunit of K_V_11.1 [3]. LQT 3, on the other hand, is a result of the gain-of-function variant in the *SCN5A* gene, encoding Na_V_1.5, which causes persistent inwards sodium flow during the plateau phase of the action potential, resulting in delayed repolarization [3, 25]. Under normal conditions, sympathetic and parasympathetic activity results in overall augmentation of depolarisation and repolarisation kinetics. However, in patients with LQTs, this can lead to prolongation of action potential duration, promoting early after-depolarization (EAD) that may trigger TdP. The three undisputed genes *KCNQ1*, *KCNH2*, and *SCN5A* account for approximately 90% of gene-positive cases, with gene-specific triggers such as exercise (LQT 1), emotional stress (LQT 2) and sleep (LQT 3), identified respectively [26].

The 3 LQT-associated CALM variants present with atypical features. In addition to QTc prolongation, CALM variants manifest in infancy or early childhood with marked sinus bradycardia or atrioventricular block, seizures and development delay (Fig. 1) [22]. Triadin (*TRDN*) is a critical protein in the cardiac calcium release complex, binding to type 2 ryanodine receptor (RyR2), calsequestrin 2 (Casq 2), junctophilin 2 (Jph 2) to release calcium from the sarcoplasmic reticulum to ensure proper excitation-contraction coupling in the heart [27]. The loss of TRDN can result in a particularly malignant phenotype, termed Triadin knockout syndrome, usually presenting in childhood [28]. These patients exhibit transient QTc prolongation and T-wave inversion in precordial leads V_3_–V_5_, that are very atypical (Fig. 1) [28]. They also display exercise-induced ectopy at peak exertion, a hallmark of catecholaminergic polymorphic ventricular tachycardia (CPVT) rather than LQTs. This overlap of clinical features has led to its consideration as a distinct disorder however, given that QT prolongation was considered the most discernible abnormality, it is currently included as an atypical phenotype of LQTs [22]. The role of *CALM 1–3* and *TRDN* genes remains to be established in typical adult LQT patients [4, 22].

**Fig. 3.**
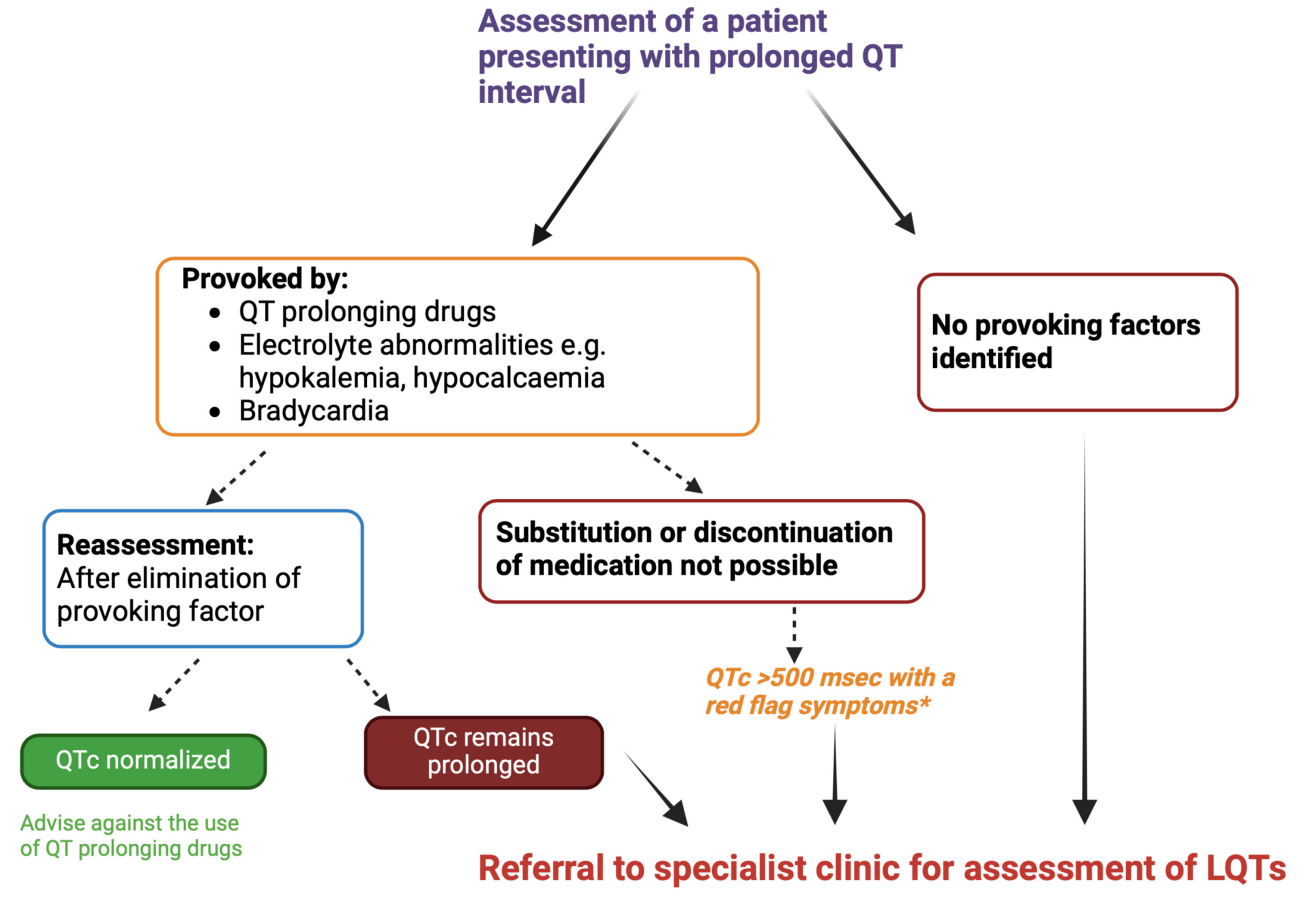
**Assessment of patients presenting with prolonged QT interval**. *Red flag symptoms include the history of syncope or a family history of unexplained sudden death. If QTc interval <500 msec with no other risk factors, reasonable to continue on medications with close monitoring of QTc interval. Fig. 3 was drawn using BioRender.

Genetic testing should be performed in any patient presenting with a prolonged QTc on the ECG. When a pathogenic mutation is found, subsequent cascade screening of family members of the proband is critical [4]. Genetic testing allows mitigation of gene-specific triggers and guides gene-specific treatment for different LQTs subtypes, which are discussed later in this review [29]. Given its high sensitivity, with a potential pathogenic variant identification in 75–80% of cases, genetic testing may similarly be considered useful in ruling out the presence of an inherited aetiology in patients where the clinical diagnosis is very borderline [30]. It is important to note, however, that in patients who are genotype-negative, but phenotype positive with clear QT prolongation, the risk of cardiac events remains equivalent, underscoring the continued need for these patients to be followed in a specialized cardiogenetics clinic and the importance of continuing β-blockers [31]. Finally, in patients where there is suspected drug-induced long QT, an underlying mutation is noted in approximately 30% of cases (Fig. 3) [32].

## 4. Artificial Intelligence in the Diagnosis and Risk Stratification of LQTs

Currently, the diagnosis of LQTS, as described initially, hinges on accurate measurement of the QTc interval and 25% of patients have ‘concealed’ LQTs (i.e., gene positive, phenotype negative), which could delay appropriate therapy, making it critical for an efficient diagnostic tool for screening and detection. AI offers a promising new means of enhancing ECG interpretation [33].

Neural networks, which are a subtype of supervised machine learning algorithms, are capable of processing large volume data, in this case, ECGs, extracting meaningful patterns such as QTc interval and T-wave morphology [34, 35, 36]. Further to this, deep learning models excel in making predictions based on these learned patterns and can assist clinicians in risk stratification and decision-making [33].

In addition to 12-lead ECG, which is commonly used in training AI models, single-lead mobile ECGs have also yielded comparable results [37, 38]. A prospective study that assessed smartwatch single-lead ECG with conventional 12-lead ECG showed negligible difference (mean QTc interval was 407 ± 26 msec on 12-lead ECG vs 407 ± 22 msec on single-lead ECGs) with 98.2% of patients having <50 msec difference between the two measures [37]. This enables the use of smartwatches and activity trackers, which can lead to patient-directed management with the potential to implement timely interventions to mitigate the risk of arrhythmias [33].

AI has the potential to transform the way we approach LQTs, both in diagnosis and risk stratification, however, we need to be cognizant of the potential pitfalls of this technology. Potential bias in data gathering and the information used to train machine learning algorithms, challenges related to the interpretability of prediction models, potential utilisation of patient information and privacy leaks are a few concerns regarding the widespread use of this technology which needs to be addressed [33].

## 5. Risk Stratification

The advent of genetic testing has led to a significant increase in the identification of patients with ‘concealed’ LQTs [9]. These individuals have a substantially lower risk of arrhythmic events, approximately eight times lower, compared to those who are both genotype and phenotype-positive patients [9]. The annual rate of sudden death in symptomatic patients, however, is approximately 5%, with a further 10-year mortality rate as high as 50% [39].

The 1-2-3-LQTS-Risk model, used in the latest European Society of Cardiology (ESC) guidelines, estimates an individual risk of life-threatening arrhythmias based on two key prognostic determinants: the QTc interval and genotype (LQT1, LQT, LQT3) [4]. Furthermore, they also reported an incremental life-threatening arrhythmic risk of 15% for every 10 msec increase in QTc interval duration and subtypes LQT 2 and LQT 3 conferring a greater arrhythmic risk compared to LQT 1 at 130% and 157% respectively [9]. This study also highlighted the commencement of β-blockers, specifically nadolol, resulted in a 62% risk reduction of life-threatening arrhythmic events.

**Fig. 4.**
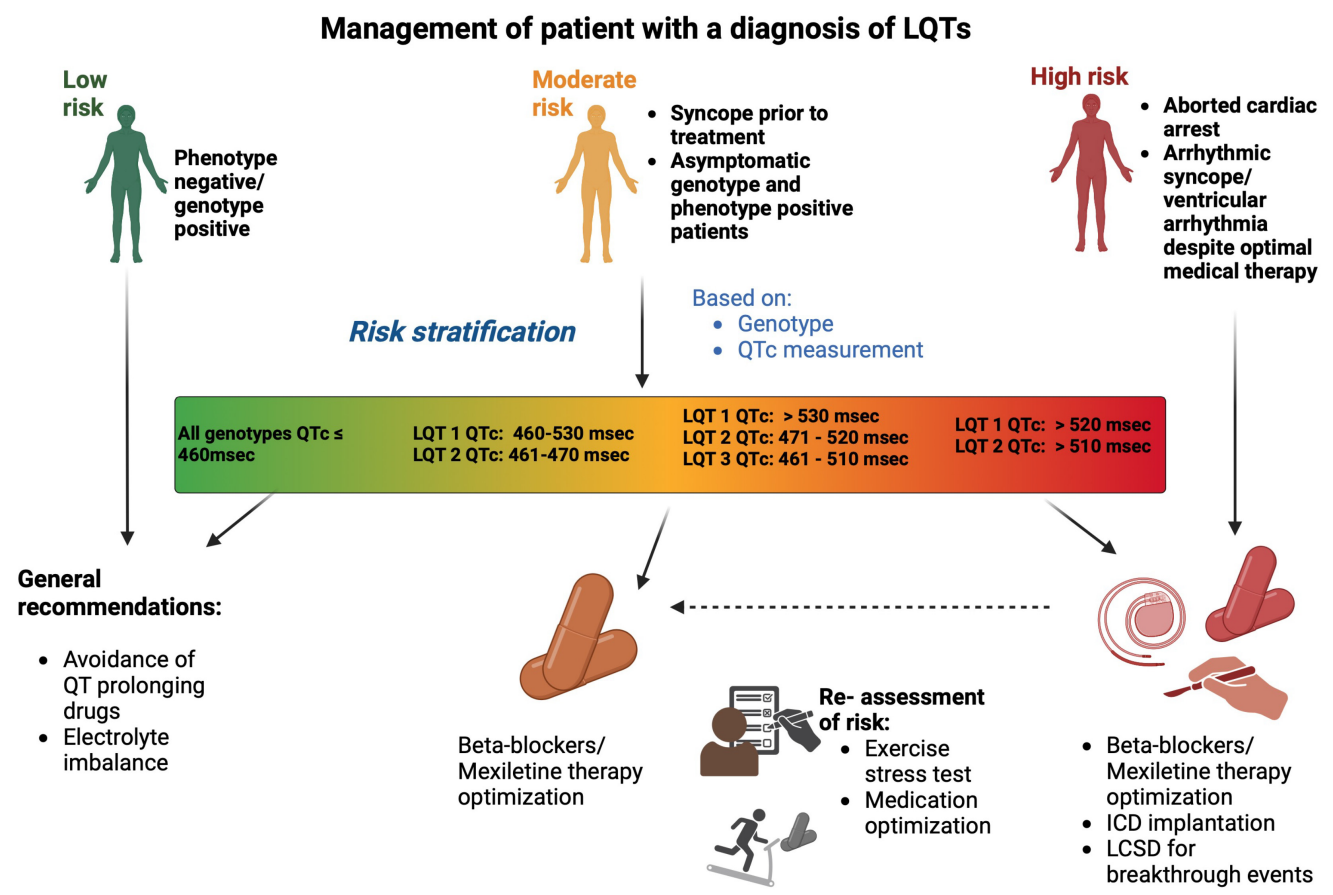
**Management of a patient with a diagnosis of LQTs**. Risk stratification is based on QTc interval and genotype, categorizing patients into low, moderate, or high-risk. Management includes avoidance of QT-prolonging drugs, and pharmalogical management with β-blockers and mexiletine. In high-risk patients, further interventions such as ICD implantation or LSCD for breakthrough arrhythmic events may be required. Ongoing risk re-assessment is critical as the clinical status and risk profile of patients may evolve. ICD, implantable cardioverter-defibrillator; LCSD, Left Cardiac Sympathetic Denervation. Fig. 4 was drawn using BioRender.

This highlights the importance of adopting a ‘dynamic’ score in patients with LQTs, given how QTc interval and its associated risk change during patient follow-up and with therapy initiation and optimisation [29]. While the 1-2-3-LQTS-Risk model represents a ‘static’ score, i.e., a score determined at the time of diagnosis, recent evidence highlights the potential for risk modification over time [40]. A study reported the ‘static’ M-FACT score of ≥2 reduced to <2 during follow-up in almost half the patients largely with β-blockers commencement [41]. It resulted in QTc interval shortening and resulted in fewer ICD implantations without increasing the rate of life-threatening arrhythmic, highlighting the importance of revisiting risk stratification in LQTs patients. A brief diagram is provided outlining the management of patients with a diagnosis of LQTs (Fig. 4).

## 6. Management

All patients with LQTs, regardless of ECG manifestation of QTc prolongation, should be given advice regarding avoidance of medications that prolong QTc interval, ensuring communication with their pharmacist. Re-iteration of the importance of adherence to beta blocker use, and avoidance of electrolyte imbalances such as hypokalaemia, is necessary at each follow-up appointment [4].

### 6.1 Medical Therapy

Drug therapy with β-blockers remains the mainstay for the prevention of arrhythmic events in patients with LQTs [4, 42, 43, 44]. Schwartz *et al*. [45] first reported the benefits of β-blockers in a patient with LQTs in 1964; this was subsequently followed by a small study that reported a dramatic reduction in mortality, from 73% to 6%, in 203 symptomatic patients with LQTs [45]. The β-blockers themselves do not result in a shortening of the QTc interval, but rather their predominant mechanism is to avoid rapid heart rates which predispose to ventricular arrhythmia.

The choice of β-blockers is critical. There is evidence to show that non-selective β-blockers, such as nadolol and propranolol, confer greater protection compared to β1 selective agent, such as atenolol and metoprolol [9, 46, 47]. While β1 adeno-receptors predominantly mediate cardiac chronotropic and inotropic response, β 2/3 of adeno-receptors, which make up 20–30%, play a significant physiological role via similar pathways [48]. A study on human atria revealed that blocking β1 adeno-receptor led to cross-sensitization and upregulation of β2 adeno-receptors. Therefore non-specific β-blockade is recommended to mitigate this risk of receptor-specific sensitisation [49, 50].

Additionally, nadolol has been shown to reduce the risk of life-threatening arrhythmias by 62% when compared to other β-blockers [9]. Despite its chemical similarity to propranolol, nadolol does not undergo first-pass degradation in the liver, thereby maintaining a low variability in plasma concentration [51, 52]. Further, the hydrophilic nature of nadolol seemingly results in fewer central nervous system side effects, making it better tolerated [52]. Both nadolol and propranolol also have an additional membrane-stabilizing effect by peak Na^+^ current blockade [53]. It remains critical to introduce β-blockers at a low dose with gradual up-titration to minimize intolerance, aiming for an optimal dose of nadolol between 1–1.5 mg/kg/day or maximal-tolerated dose in adult patients with LQTs [43]. If propranolol is considered, a dose of 2–3 mg/kg/day with some patients requiring up to 4 mg/kg/day [54]. The dose of the β blocker should be evaluated at each interval follow-up with q6-month to yearly exercise stress tests. It has now become clear that metoprolol should not be the first line in the management of LQTs and there is limited data available on the use of atenolol [9, 54].

Another common concern is the use of β-blockers during pregnancy. Well-intentioned practitioners may elect to discontinue non-selective β-blockers to avoid neonatal adverse effects such as intrauterine growth restriction, hypoglycaemia and bradycardia [55, 56]. However, a retrospective study which assessed the use of β-blockers in 153 women with LQTs, showed an 80% risk of cardiac events in the postpartum period, especially in LQT 2, with β- blocker discontinuation [56]. In contrast to this, women who were prenatally diagnosed and commenced on β-blockers, had no cardiac events, underscoring the importance of continued use of β-blockers during pregnancy. Importantly, there were comparable rates of intrauterine growth restriction between nadolol and other cardio-selective β-blockers use, at 33% vs 40% (*p* = 0.9) respectively [57]. As such, β-blockers remain ‘first-line’ therapy in women with LQTs. Switching well managed women with LQTs on nadolol to metoprolol, due to concerns for fetal well-being is strongly discouraged, as this could lead to fatal outcomes, especially in high-risk patients such post-partum women with LQT 2 [58].

Despite its efficacy, adherence to β blocker therapy remains a concern, with a mere 40–50% compliance rate reported in some studies [59, 60]. This has important implications for the management of arrhythmic events and educating patients on adherence remains crucial.

### 6.2 Mexiletine for LQT 3

LQT 3 can lead to a risk of life-threatening arrhythmias secondary to bradycardia [61]. Unlike other LQTs where ventricular arrhythmia occurs secondary to sympathetic activation, in this unique bradycardia-dependent QTc prolongation, the traditional use of β-blockers has been questioned [62]. However, recent evidence has demonstrated the effectiveness of nadolol in all subtypes of LQTS, including LQT 3 [9, 63].

Mexiletine is a class 1b anti-arrhythmic agent that can suppress the effects of I _Na-L_, which has a ‘gain-of-function’ effect of SCN5A mutation in patients with LQT 3. Mexiletine has demonstrated shortening of the QTc interval by 63 ± 6 milliseconds, with 73% of patients achieving a QTc interval reduction below the high-risk threshold. Additionally, 60% of patients attained a QTc interval value within the ‘normal’ range after starting mexiletine, and a three-year follow-up demonstrated a 93% suppression of cardiac events in these patients [9].

However, the extent of QTc shortening with mexiletine strongly correlates with the baseline QTc interval and may not always lead to the suppression of arrhythmic events [64, 65]. Current ESC guidelines suggest verifying QTc interval shortening by at least 40 msec before long-term prescription of the agent, given the differential responses to mextiline in patients with LQT 3 [4]. Ranolazine, an anti-anginal agent and eleclazine, a novel selective inhibitor of I _Na-L_, have shown promising outcomes in shortening QTc interval in LQT 3, and clinical testing of these medications is currently underway [66, 67].

### 6.3 ICDs

Patients with a history of cardiac arrest or ventricular arrhythmias should have an ICD implanted, given a 14% recurrence risk within 5 years [68]. Those with a clear diagnosis of LQTs and a history of malignant syncopal episodes, despite initiation of β-blockers, are similarly recommended to undergo implantation [4, 44]. Furthermore, in high-risk asymptomatic patients, by 1-2-3-LQTS Risk profile, ICD implantation may be considered in addition to genotype-specific medical therapy [4]. This underscores the importance of treatment optimization in high-risk individuals, such as a patient with LQT 2 with QTc interval >500 msec, rather than equating high-risk to an ICD implantation [40].

The choice regarding a transvenous system as opposed to a subcutaneous ICD predominantly depends on the need for pacing support. A subcutaneous ICD consists of a parasternal subcutaneous lead connected to an active pulse generator positioned in the axillary region, with the entire system placed extravascularly [69]. This design reduces the risk of systematic infection and preserves the vascular system which is an important consideration in young patients with inherited cardiac conditions who may face long-term device-related complications [63, 64]. This is an important consideration when choosing the device, especially in patients where arrhythmias may be triggered by pause or bradycardia may be a limiting factor in β- blocker dose optimisation [70]. As such careful evaluation of the patient’s age, phenotype and genotype are critical considerations before device implantation, where indicated.

### 6.4 Left Cardiac Sympathetic Denervation (LCSD)

The role of cardiac sympathectomy in LQTs was first reported by Moss and McDonald in 1971 [71]. Although the exact mechanism of neuromodulation in LQTs remains to be completely elucidated, it reduces levels of norepinephrine release, thereby raising the threshold for ventricular fibrillation [72]. In addition to the therapeutic effects of beta-blockade, LCSD has an additive alpha-adrenergic-mediated effect due to preganglionic denervation that prevents repolarisation heterogenicity [73].

Initial studies reported LCSD to be effective in patients who remained symptomatic or presented with cardiac arrest despite the use of β-blockers. A recently published large retrospective with a 50-year follow-up of 125 consecutive patients who underwent LCSD for LQTs demonstrated it to be both efficacious and safe [74]. LCSD resulted in an 86% decrease in the mean yearly cardiac events, with 17% of a very high-risk subgroup of patients remaining asymptomatic. In addition to this, where the initial QTc interval exceeded 500 msec, they reported an average QT reduction of 60 msec in half of these patients [74].

The Heart Rhythm Society/European Heart Rhythm Association/Asia Pacific Heart Rhythm Society consensus document recommends LCSD in symptomatic patients despite the use of β-blockers when ICD is contra-indicated or refused, as well as in patients who have multiple shocks despite the use of β-blockers [4]. However, given that some patients still experience breakthrough events after the procedure, LCSD monotherapy is not recommended as an alternative to ICD implantation in high-risk patients [75].

## 7. Future Therapies

### 7.1 Catheter Therapy

Unlike structural cardiomyopathies, such as hypertrophic cardiomyopathy and arrhythmogenic cardiomyopathy, primary electrical conditions, such as LQTs, Brugada and catecholaminergic polymorphic ventricular tachycardia, result from ion channel abnormalities. Advanced cardiac imaging tools, such as speckle tracking, have shown subtle electrical and anatomical abnormalities in primary electrical conditions [76]. In initial electroanatomical mapping studies, more than 2 decades ago, Haïssaguerre *et al*. [77] demonstrated that in LQTs, premature ventricular contractions (PVCs) originated from the Purkinje system of the left ventricular, while in Brugada syndrome, the PVCs originated from the right ventricular outflow tract (RVOT). Following this, in Brugada syndrome, bipolar low-voltage areas and abnormal late potentials were identified in the RVOT with ablation in this region resulting in the normalisation of the Brugada pattern on the surface ECG [78].

Pappone *et al*. [79] sought to reveal similar findings in LQTs. They observed abnormally prolonged and fragmented electrograms in the RVOT of patients with LQTs, similar to that seen in with Brugada syndrome. Radiofrequency ablation of these abnormal substrates in the RVOT showed a reduction in ventricular arrhythmia burden and shortening of QT interval, suggesting that epicardial right ventricular outflow tract modification may be beneficial for patients with LQTS. The right ventricular epicardium (and particularly the RVOT) has a lower expression of connexins and cardiac sodium channels, which heightens its vulnerability to cardiac arrhythmias compared to other myocardial regions [80]. Although fragmentation of electrograms arising from this region has been postulated as the mechanism for the arrhythmogenic substrate, the precise mechanism of these abnormalities needs further clarification [81]. Larger, well-characterized controlled studies are required to explore this interesting preliminary observation, given the genetic and phenotypic heterogeneity presentations of LQTs [82].

### 7.2 Gene Replacement Therapy

Current therapies in LQTs primarily target symptoms to reduce arrhythmia-triggering events but fail to address the underlying molecular cause (i.e., ion channel dysfunction). Gene therapy promises mechanism-driven therapy. At present, gene replacement therapy, gene silencing therapy and direct genome editing are the three main means of achieving gene therapy [83].

In gene replacement therapy, pathogenic variants that result in insufficient production of protein, are the primary target [84]. Therapy is aimed at either augmenting or correcting the expression of the deoxyribonucleic acid (DNA) sequence to ensure near normal production of the protein. In gene silencing therapy, the main aim of therapy is to inactivate the mutated DNA sequence, which often produces adequate protein but exhibits harmful functionality that results in a dominant negative effect [84]. These 2 mechanisms of therapy are achieved through ribonucleic acid (RNA)-based strategies, with variable exogenous protein-coding sequence delivery, either a viral vector (such as adeno-associated virus 9) or via lipid nanoparticles [85, 86].

A recent proof-of-concept study in LQT 1 rabbits demonstrated a pronounced shortening of action potential duration with KCNQ1-Suppression Replacement (SupRep) gene therapy when compared to LQT 1 controls (LQT1-Untreated vs LQT1-SupRep, *p* < 0.0001, LQT1-SupRep vs wild type = non-significant) [83]. This novel hybrid therapy combines both gene silencing and gene replacement into a single ‘suppression-and-replacement’ gene therapy. This hybrid therapy combines a single construct of KCNQ1-shRNA (suppression) and a shRNA-immune (shIMM) KCNQ1-cDNA (replacement), packaged into adeno-associated virus serotype nine and delivered *in vivo* via an intra-aortic injection. The SupRep-treated rabbits demonstrated a pronounced (13 ± 4%) shortening of the QT index and the action potential duration 90 (394 ±15 msec), bringing both parameters near the level of wild-type rabbits. These SupRep-treated rabbits, when subjected to adrenergic stimulation with isoproterenol (with a 20% increase in heart rate), behaved nearly identical to wild-type rabbits (QT index 16.5 vs 16.9). This has prompted the development of a KCNH2 SupRep construct, designed as a potential treatment for LQT 2 patients, with initial safety and efficacy trials currently underway in rabbit models with KCNH2-mediated LQTs [87, 88].

These RNA-based therapies are opposed to direct genome editing, in which the DNA sequence is modified with permanent effects. One such tool that has gained traction recently is the clustered regularly interspaced short palindromic repeats (CRISPR)/Cas9 nuclease system [84]. CRISPR/Cas 9 results in double-strand breaks in DNA, generated by programmable nuclease, with DNA sequences which can be inserted or deleted to inactivate an abnormal gene copy either *in vitro* or *in vivo* [89]. Its use is currently being studied, *in vivo*, on patient-derived induced pluripotent stem cells [90, 91]. Base and prime editors which comprise Cas9 nickase and a modified reverse transcriptase, can introduce, delete or substitute nucleotides without DNA breakage and are considered to have a more specific effect [84]. This method has been studied, in which a mouse with a pathogenic variant of SCN5A resulting in LQT 3, was effectively treated by the delivery of adenine base editor delivery via an adeno-associated virus vector [92].

However, off target mutations as well as tissue specific delivery of genome editors remain a hurdle. Another important concern regarding genome editing is the heritability of editing, as in theory genome editing can be expanded to germline cells (i.e., in human embryos) to treat inherited cardiac conditions such as LQTs with serious ethical issues [89].

Clinical genetic therapy holds the promise of personalised therapy in cardiac genetics. The presence of numerous unique LQT-causing variants and the complexity of cofounding mechanisms make these current methods challenging for widespread clinical use at present.

## 8. Discussion 

Despite enhanced understanding, LQTs continue to pose a diagnostic and management challenge in everyday clinical practice. While the identification of key pathogenic variants and development of subtype-specific treatment strategies have significantly improved patient outcomes, many patients remain underdiagnosed or misdiagnosed due to limitations of current diagnostic tools especially those with concealed or borderline phenotype [7]. Overdiagnosis also remains a concern, as misinterpretation of QTc interval can lead to unnecessary interventions, such as ICD implantation and long-term medication use [8].

In the era of technology, AI-enabled tools offer a promising solution to the detection of subtle ECG abnormalities, through deep learning algorithms [93]. Furthermore, the intergration of AI with wearable technologies, such as smartwatch-based single-lead recordings, could further facilitate timely diagnosis empowering patient-directed monitoring [37, 38]. However, the inherent challenges such as data validation across a diverse population, transparency in algorithm-based decision making and concerns regarding data privacy need to be addressed before the widespread adoption of these tools [33].

In addition to early detection and diagnosis, the forefront of management for LQTs is also shifting to genotype-guided management. Recent interest in gene replacement therapies, such as the SupRep and CRISPR, offers an opportunity for precision-based medicine in the near future.

## 9. Conclusion

LQTs is an inherited cardiac condition which occurs as a result of maladaptive cardiac repolarisation. It is imperative that physicians assess the patient’s clinical presentation, family history, and genetic test results in conjunction with QT measurement on ECG. Although a missed diagnosis of LQTs can be fatal, erroneous overdiagnosis of LQTs can also lead to substantial lifelong implications.

In the era of precision medicine, there is a paradigm shift in the clinical care of these patients, with the possibility of delivering patient-centric therapy in the near future.
